# Handy-Book of the Treatment of Women’s and Children’s Diseases, Etc.

**Published:** 1871-10

**Authors:** 


Handy-Book of the Treatment of Women's and Children's Diseases, according to the Vienna Medical School, with Prescriptions. By Dr. Emil Dilluberger. Translated from the second German edition, by Patrick Nicol, M. B. Philadelphia: Lindsay & Blakiston.

In this little volume we have presented, in a condensed form, a considerable number of valuable suggestions and directions in regard to the treatment of women's and children's diseases.

